# Regulation of Sirtuin 3-Mediated Deacetylation of Cyclophilin D Attenuated Cognitive Dysfunction Induced by Sepsis-Associated Encephalopathy in Mice

**DOI:** 10.1007/s10571-017-0476-2

**Published:** 2017-02-25

**Authors:** Fan Sun, Yanna Si, Hongguang Bao, Yajie Xu, XiaoXiao Pan, Lingqing Zeng, Ling Jing

**Affiliations:** Department of Anesthesiology, Nanjing First Hospital, Nanjing Medical University, Nanjing, 210006 Jiangsu China

**Keywords:** Cognitive dysfunction, Cyclophilin D, Deacetylation, Mitochondrial permeability transition pore, Sepsis-associated encephalopathy, Sirtuin 3

## Abstract

The present study aimed to investigate cognitive dysfunction in the hippocampus induced by sepsis-associated encephalopathy (SAE) via acetylation of cyclophilin D (CypD) and opening of mitochondrial permeability transition pore. It also explored whether activating sirtuin 3 (SIRT3) can mediate deacetylation of CypD and prevent the development of SAE. Male mice were randomly assigned to six groups: sham group, cecal ligation puncture group, CypD siRNA transfection (CypD-si) group, CypD control siRNA transfection (CypD-c) group, SIRT3 overexpression vector pcDNA3.1 (SIRT3-p) group, and SIRT3 empty vector pcDNA3.1 (SIRT3-v) group (*n* = 18). The CypD-si and CypD-c groups were transfected with CypD siRNA and CypD control siRNA, respectively. The SIRT3-p and SIRT3-v groups were injected with SIRT3 pcDNA3.1 and vector pcDNA3.1, respectively. The learning and memory function was assessed using the learning version of the Morris water maze test. Then, cell apoptosis and the levels of CypD, acetylated CypD, SIRT-3, interleukin 6 (IL-6), tumor necrosis factor-α (TNF-α), and caspase-3 in the hippocampus were determined. The levels of CypD and acetylation of CypD increased in the hippocampus induced by SAE. Increasing SIRT3 and decreasing CypD can attenuate cognitive impairment and neuroapoptosis, and protect the integrity of mitochondrial membrane from damage and restore the protein expressions of IL-6, TNF-α, and caspase-3. Activating SIRT3-mediated deacetylation of CypD attenuated learning and memory dysfunction induced by SAE.

## Introduction

It is universally acknowledged that severe sepsis is related to multisystem damage including brain dysfunction, and sepsis-associated encephalopathy (SAE) is one of the severe central nervous system complications in patients in intensive care units for its cognitive dysfunction (Cotena and Piazza 2012). SAE is associated with increased morbidity and mortality, and it can influence the short-term outcome and long-term recovery of patients with SAE (Cotena and Piazza 2012; Widmann et al. 2014).

Of most importance to the pathogenesis of SAE is the mitochondrial dysfunction (Wu et al. 2015; Bozza et al. 2013). The involvement of mitochondria is far beyond energy production. It is involved in diverse metabolic pathways and also have significant effect for cells and the function of the organism (Zampieri et al. 2011). A large amount of evidence indicates that mitochondrial dysfunction induced by SAE follows the opening of mitochondrial permeability transition pore (mPTP) (Comim et al. 2008). Cyclophilin D (CypD), an apoptosis-regulated protein, locates in the mitochondrial matrix, which can be translocated to the inner mitochondrial membrane, and plays an important role in regulating mPTP opening (Kim et al. 2014).

Previous studies also showed that sirtuins, which are part of nicotinamide adenine dinucleotide (NAD^+^)-dependent deacetylases, can regulate the acetylation of protein (Hill et al. 2009). Sirtuins are regarded as “metabolic sensors,” which can encode seven sirtuin isoforms: SIRT1–SIRT7. SIRT3 is studied the most with its location in the mitochondria (Houtkooper et al. 2012; Lombard et al. 2007). The evidence points out that CypD can be deacetylated by SIRT3, which results in inhibiting mPTP opening (Hafner et al. 2010). Activating SIRT3 can attenuate mitochondrial dysfunction and neurological impairment after traumatic brain injury in aged mice (Wang et al. 2016). We know that CypD can facilitate the opening of mPTP and trigger synaptic degeneration, leading to Alzheimer’s disease (Du et al. 2014). However, the effects of SIRT3-mediated deacetylation of CypD in cognitive impairment induced by SAE remain unknown.

This study aimed to investigate cognitive dysfunction in mice induced by SAE via acetylation of CypD and promotion of mPTP opening. Activating SIRT3 can mediate the deacetylation of CypD and prevent the development of SAE. This study also opens new perspectives for developing preventive therapies against the development of cognitive dysfunction induced by SAE.

## Methods

### Animals

The experimental procedures were approved by the animal ethical committee of the Nanjing Medical University, China, and performed in strict accordance with the National Institutes of Health *Guide for the Use of Laboratory Animals*, and all efforts were made to minimize animal suffering. Male C57BL/6J mice (age 3–4 months), weighing 20–30 g, were housed in controlled cages (temperature 22–24 °C and 60–65% humidity) with a 12-h light–dark cycle at the Animal Experiment Centre, Nanjing Medical University, China. The mice were randomly assigned to six groups: sham group, cecal ligation puncture (CLP) group, CypD siRNA transfection (CypD-si) group, CypD control siRNA transfection (CypD-c) group, SIRT3 overexpression vector pcDNA3.1 (SIRT3-p) group, and SIRT3 empty vector pcDNA3.1 (SIRT3-v) group (*n* = 18).

### CypD siRNA and Transfection

The siRNA against mouse CypD was synthesized by Nanjing Kaiji Biotech (Nanjing, China). The pairs were: 5-ACACCAATGGCTCTCAGTTC-3 (forward) and 5-AGTGGCCTTCCTCATACTCA -3 (reverse) (Hu et al. 2015). The entranster-in vivo transfection reagent (18668-11) was purchased from Engreen Biosystem Co, Ltd, China. The Entranster-in vivo–siRNA mixture was prepared strictly in accordance with the manufacturer’s instructions. The mice were well tolerated with 50 μL siRNA infusion, and no signs of neurotoxicity, including vocalization, food intake, hind-limb paralysis, or damage of the nervous system, were observed in the preliminary study. The CypD-si and CypD-v groups were injected into mice lateral cerebral ventricles with CypD siRNA and CypD control siRNA, respectively, 3 days before surgery.

### Plasmids and Transfection

The SIRT3 overexpressing vector and the empty vector were purchased from Dharmacon Research Inc. (Lafayette, CO, USA). The SIRT3-p and SIRT3-c groups were injected into mice lateral cerebral ventricles with SIRT3-Flag and Vec, respectively, 3 days before surgery.

### Surgical Procedure

All animals were subjected to CLP surgery as described previously (Rittirsch et al. 2009). Each mouse in all groups was anesthetized with intraperitoneal injection of 2% sodium pentobarbital (40 mg/kg, Sigma, USA) before surgery. The cecum was identified and ligated with 4.0 silk at the ileocecal junction from the distal end. Then, the cecum was punctured with a 22-gauge needle at the same point, and the contents were squeezed into the abdominal cavity lightly. Then, the abdomen was closed by simple suture. In the sham group, the cecum of mouse was isolated in the same way as mice in the other groups but was neither ligated nor perforated. All animals were subcutaneously injected with lactated Ringer’s solution (30 mL/kg) immediately after the surgery for supplement and were then returned to their cages with warm cotton pad and allowed free access to food and water.

### Morris Water Maze

The Morris water maze (MWM), which used the computerized video track system (Smart Junior Software, Panlab, Spain) as described previously (Shin et al. 2014), assessed the learning and memory function of mice. In training phase, all animals were trained for 3 consecutive days with three trials—each one was trained for finding platform with 60 s maximum and 30 s on the platform per trial. If the mice could not reach the platform within 60 s, they would be guided to stay there for 15 s. The data of latency (time to reach the platform) were recorded by a video tracking system (Logitech, Nanjing, China). The platform was removed for the probe trail on the fourth day. All mice were monitored for 60 s in order to observe the distance of swimming and record the time spent in the target quadrant.

### Apoptosis Assay

The terminal transferase dUTP nick-end labeling (TUNEL) assay was used to detect nuclear DNA fragmentation in the hippocampus. In brief, 4-μm-thick paraffin sections were deparaffinized and rehydrated, and permeabilized with proteinase K. The slides were incubated in an equilibration buffer for 20 min at 95 °C for TUNEL staining subsequently. The hippocampal tissue sections were stained using a TACS 2 TdT-diaminobenzidine In Situ Apoptosis Detection Kit (Trevigen, MD, USA) with the manufacturer’s instruction.

### Mitochondrial Membrane Potential (MMP) Level

The MMP level was measured by fluorescence intensity, which is 490 nm of excitation and 520 nm emission in a spectrofluorometer, and was performed as per the manufacturer’s instructions given along with a mitochondrial membrane potential detection kit (Genmed Scientifics Inc., USA).

### mPTP Opening

The mPTP opening leads to mitochondrial swelling, and its decrease can be detected spectrophotometrically in light scattering and absorbance at 540 nm, which was tested by a colorimetric assay kit (Genmed Scientifics Inc., USA).

### Western Blot Analysis

The protein concentration of frozen hippocampus tissue from each mouse was tested by the bicinchoninic acid (BCA) method with bovine serum albumin as the standard. The primary antibodies against CypD, acetylated CypD, SIRT-3, interleukin-6 (IL-6), tumor necrosis factor-α (TNF-α), and caspase-3 at 1:1000 dilution (Santa Cruz Biotechnology) were performed to the membranes. The band signals were normalized to the corresponding β-actin. The protein bands were detected by an enhanced chemiluminescent detective system (Amersham Biosciences UK Ltd.) and then quantitatively analyzed using the Quantity One software package (Bio-Rad Laboratories, UK).

### Statistical Analysis

All values were presented as mean ± standard deviation (SD). Statistical analysis was carried out using the SPSS 19.0 statistical software (SPSS Inc., USA). Comparisons among multiple groups were determined by one-way analysis of variance (ANOVA) followed by Tukey’s multiple comparison test where appropriate. The place trials of the MWM were analyzed using two-way analysis of variance followed by Bonferroni multiple comparison testing. Values of *P* < 0.05 were considered to indicate statistically significant difference.

## Results

### SIRT3-Mediated CypD Improved the Learning and Memory Function in Mice Induced by SAE

**Fig. 1:**
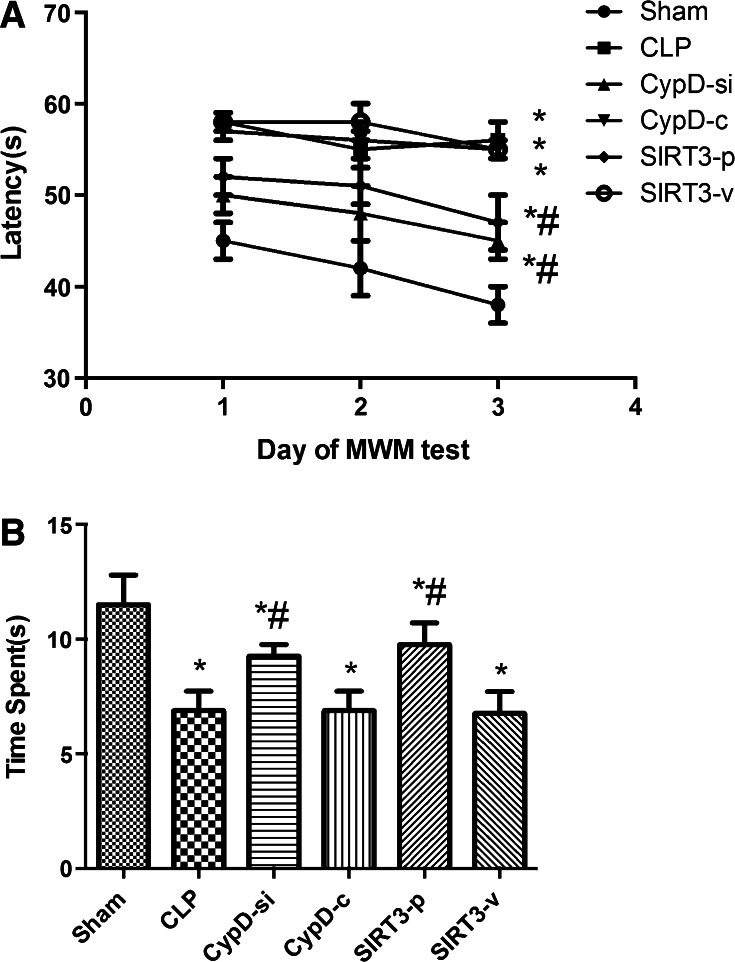
SIRT3-mediated CypD improved the learning and memory function in mice induced by SAE. **a** The latency for the mice to reach the platform was measured in spatial acquisition trials. **b** The time spent in the target quadrant was measured to assess the memory retention capabilities in the probe trial. Data are represented as mean ± SD (*n* = *18*). * *P* < 0.05, versus the sham group; ^*#*^ *P* < 0.05, versus the CLP group

In the place trial, the mice undergoing CLP surgery spent more time to find the platform than those in the sham group (*P* < 0.05). However, the mice in the CypD-si and SIRT3-p groups spent less time to find the platform than those in the CLP group (*P* < 0.05). The results indicated that CypD increased in the SAE mice, and the function of cognitive impairment might have improved when SIRT3 was inhibited (*P* < 0.05) (Fig. 1a). Swimming speed was also analyzed during the place trial, and no significant differences were observed in animals among the six groups (*P* > 0.05).

In the probe trial, the mice in the sham group spent more time in the target quadrant where the platform was located compared to those undergoing CLP surgery (*P* < 0.05). The data indicated that SIRT3 overexpression restored the spatial memory function (Fig. 1b).

### SIRT3-Mediated CypD Protected the Function of Mitochondria from Damage

**Fig. 2:**
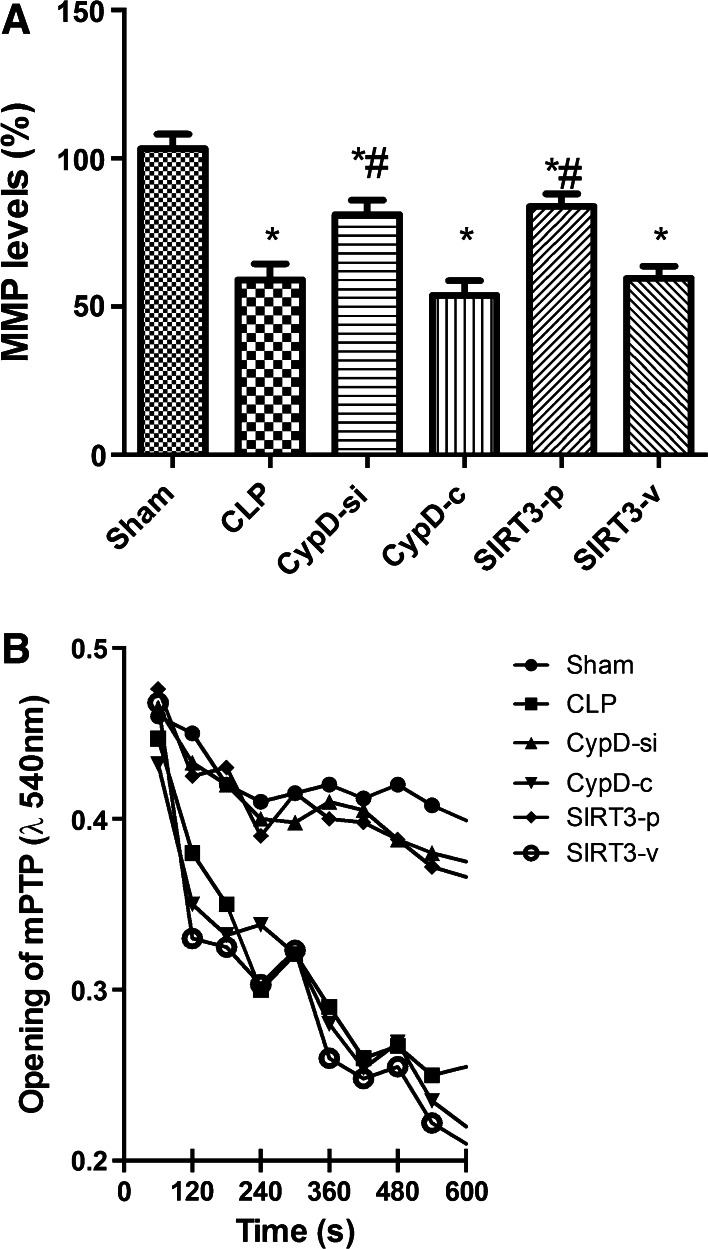
SIRT3-mediated CypD protected the function of mitochondria from damage. **a** Mitochondrial membrane potential (MMP) in the hippocampal place cells. **b** Mitochondrial permeability transition pore (mPTP) opening in the hippocampal place cells. Data are presented as mean ± SD (*n* = 5). ^*^ *P* < 0.05, versus the sham group; ^*#*^ *P* < 0.05, versus the CLP group. MMP, mitochondrial membrane potential; mPTP, mitochondrial permeability transition pore

Since an increased expression of CypD is related to a decreased threshold for mPTP opening (Naga et al. 2007), mitochondrial membrane potential (MMP) (Fig. 2a) and mPTP formation (Fig. 2b) were examined among the six groups. Compared with the CLP group, the administration of CypD siRNA and SIRT3 overexpression restored MMP level and inhibited the opening of mPTP, counteracting the adverse effect of CLP, while the CypD-c and SIRT3-v groups had no such effect.

### SIRT3-Mediated CypD Attenuated the Cell Apoptosis in the Hippocampus

**Fig. 3:**
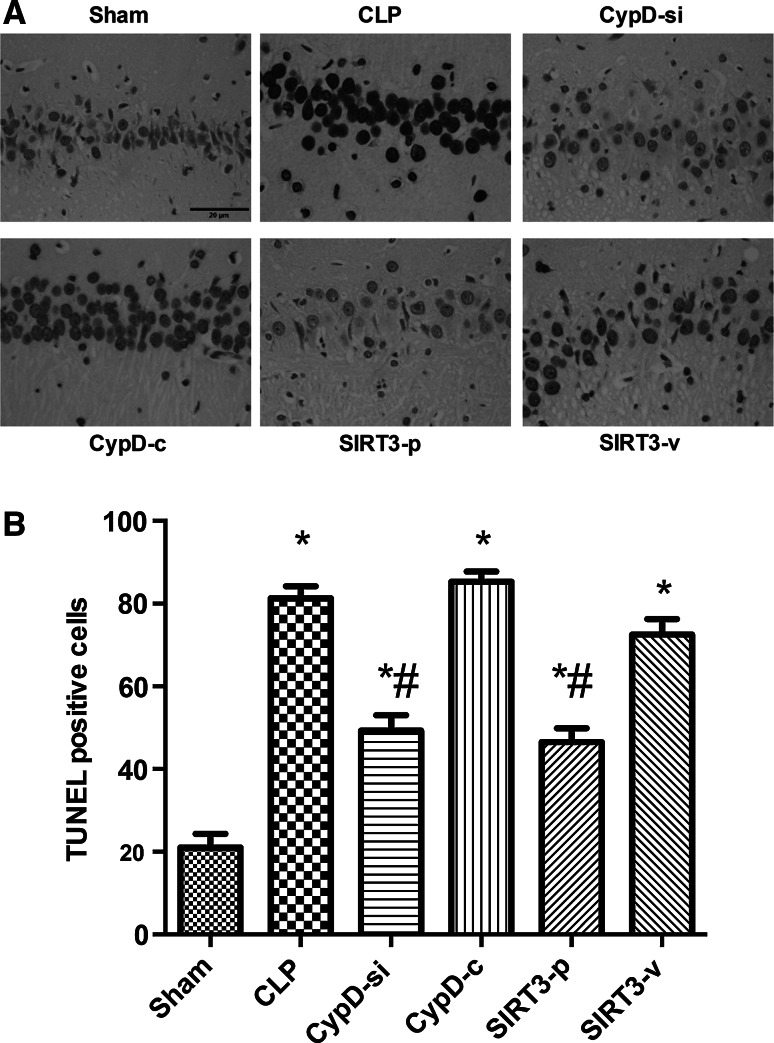
SIRT3-mediated CypD attenuated the cell apoptosis in the hippocampus. **a** Representative microphotographs were taken from the hippocampus of the Sham, CLP, CypD-si, CypD-c, SIRT3-p, and SIRT3-v groups in mice. Apoptosis was evaluated by terminal deoxynucleotidyl transferase dUTP nick end (TUNEL) staining. **b** Quantification of TUNEL positive cells in the hippocampal region were counted following MWM. *Bar* 20 μm. Data are represented as mean ± SD (*n* = 5). ^*^ *P* < 0.05, versus the sham group; ^*#*^ *P* < 0.05, versus the CLP group

The number of TUNEL-positive nuclei expressed as a percentage of total nuclei in the hippocampus increased significantly in the CLP group compared with the sham group. The increased neuronal apoptosis was partially decreased by CypD siRNA transfection or SIRT3 plasmid transfection (*P* < 0.05) (Fig. 3).

### Effects of SIRT3 Plasmid on the Expressions of IL-6, TNF-α, and Caspase-3 in the Hippocampus

**Fig. 4:**
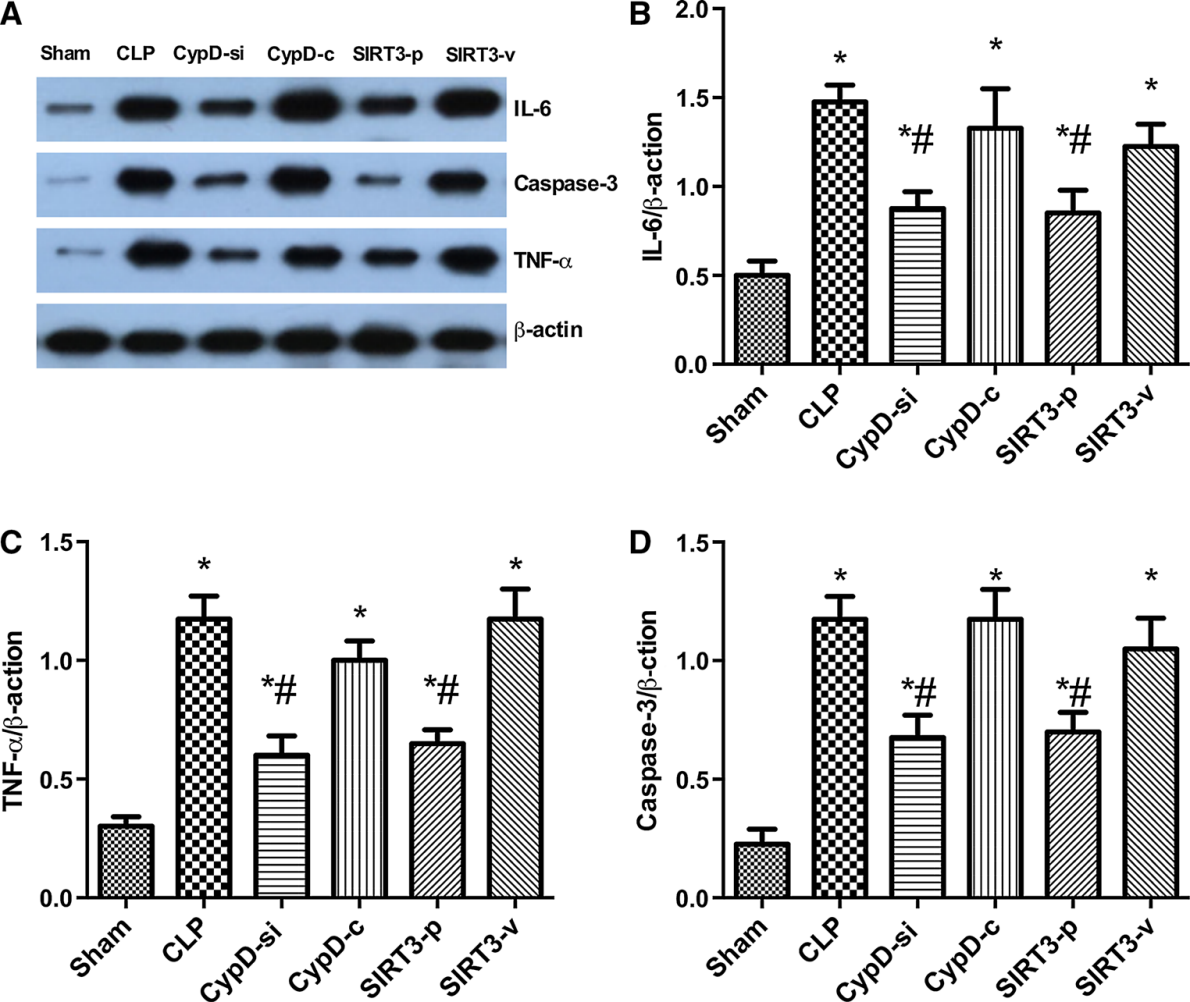
Effects of SIRT3 plasmid on the expression of IL-6, TNF-α, and caspase-3 in the hippocampus. Representative Western blots for IL-6, TNF-α, and Caspsae-3 (**a**) in the hippocampus were performed in the six groups. Densitometry analyses of Western blots for the ratio of **b** IL-6 to β-actin, **c** TNF-α to β-actin, and **d** caspase-3 to β-actin were performed. Data are represented as mean ± SD (*n* = *5*). * *P* < 0.05, versus the sham group; ^*#*^ *P* < 0.05, versus the CLP group

Western blot analysis was used to test the expressions of IL-6, TNF-α, and caspase-3 in the hippocampus of animals in six groups. The results showed that the expression of IL-6, TNF-α, and caspase-3 increased significantly in the hippocampus in the CLP, CypD-c, and SIRT3-v groups compare with the sham group. However, inhibition of CypD and the overexpression of CypD the decrease in the level of CypD and increase in the expression of SIRT3 obviously restored the alterations of IL-6, TNF-α, and caspase-3 proteins induced by SAE (Fig. 4).

### SIRT3-Mediated Deacetylation of CypD Aggravated the Progression of SAE

**Fig. 5:**
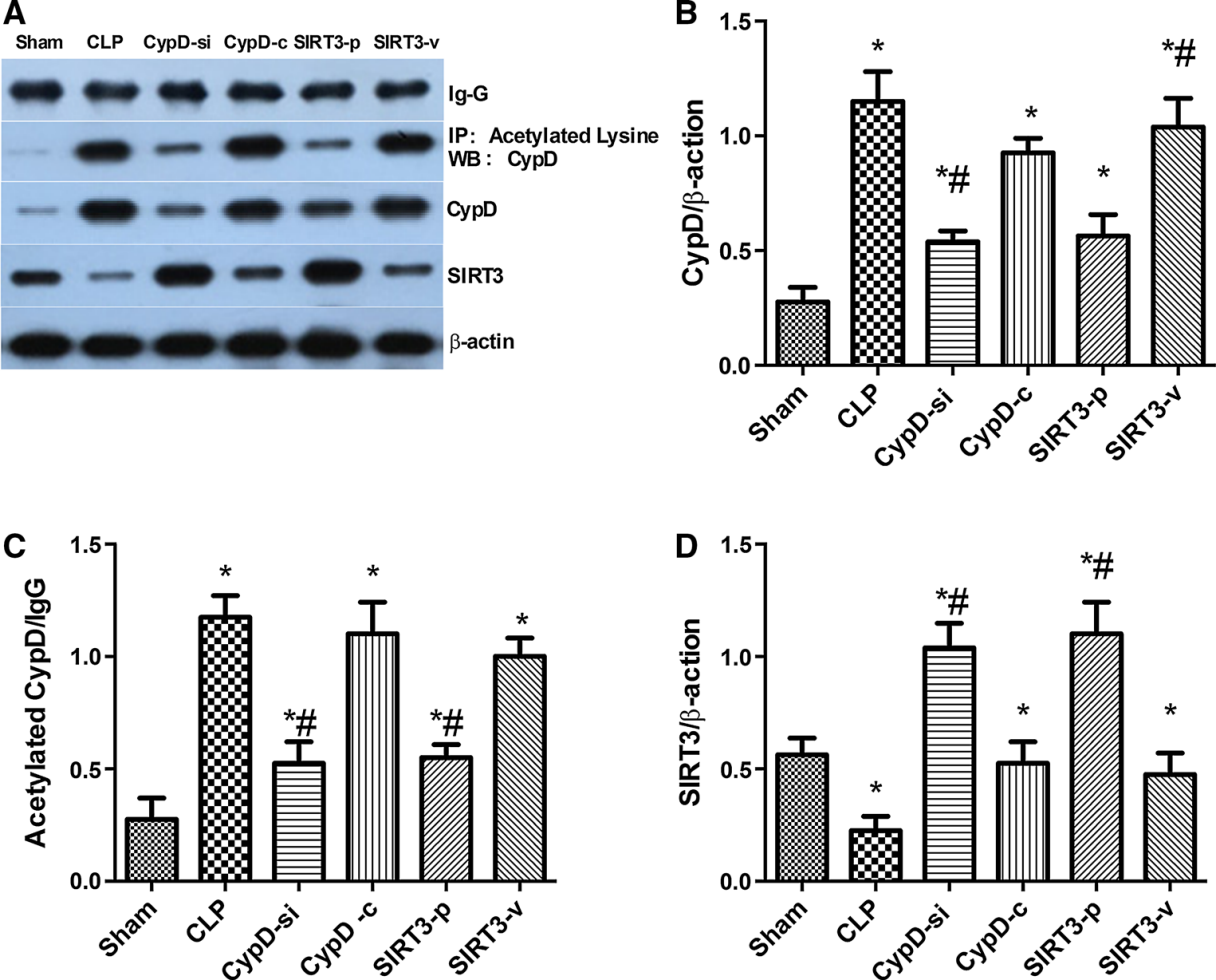
SIRT3-mediated deacetylation of CypD aggravated the progression of SAE. Representative Western blots of CypD, acetylated CypD, and SIRT3 (**a**) of the hippocampus were performed in the six groups. Densitometry analyses of Western blots for the ratio of **b** CypD to β-actin, **c** acetylated CypD to IgG, and **d** SIRT3 to β-actin were performed. Data are represented as mean ± SD (*n* = *5*). * *P* < 0.05, versus the sham group; ^*#*^ *P* < 0.05, versus the CLP group

The CypD activity increased significantly in mice which underwent CLP operation compared with the sham group (*P* < 0.05) (Fig. 5b). The decrease in the SIRT3 activity was significantly prevented in the CypD-si group compared with the CLP group (*P* < 0.05) (Fig. 5d). The expressions of total CypD protein and CypD acetylation increased after cognitive dysfunction in the hippocampus of mice in the CLP group compared with the sham group (*P* < 0.05) (Fig. 5b, c). The expressions of total and CypD acetylation decreased in the SIRT3-p group compared with the CLP group (*P* < 0.05) (Fig. 5b, c). Also, the SIRT3 levels increased in the CypD-si group compared with the CLP group (*P* < 0.05) (Fig. 5d).

## Discussion

SAE is associated with mitochondrial dysfunction, especially related to mPTP (Widmann et al. 2014; Wang et al. 2014). Also, CypD plays an important role in mPTP. SIRT3-mediated deacetylated CypD can inhibit the formation of inflammation and attenuate the mitochondrial dysfunction (Hafner et al. 2010; Marques-Aleixo et al. 2015). This study suggested that the administration of CypD inhibition and SIRT3 activation protected the learning and memory dysfunction in SAE mice.

The mice undergoing CLP surgery in this study showed that crouching, less contraction of arrector pili muscles, shortness of breath, and other symptoms were observed. At the same time, the Morris water maze experiment showed that the escape latency of the mice and the time of exploring shortened significantly in the sham group compared with the CLP group, suggesting that the sepsis model was successful. In the place trial, the mice undergoing CLP surgery spent more time in finding the platform than those in the sham group. However, the mice in the CypD-si and SIRT3-p groups spent less time in probe trail phase than those in the CLP group. The results indicated that CypD played the important role in cognitive dysfunction induced by sepsis, and SIRT3 overexpression attenuated cognitive impairment in sepsis mice. In the probe trial, the mice in the sham group spent less time in the target quadrant where the platform was located compared to those undergoing CLP. The findings indicated that inhibition of CypD expression in hippocampus alleviated the progression of SAE, and also the overexpression of SIRT3 attenuated this effect and restored the spatial memory function.

A growing number of researches indicate that sepsis can increase the expression of activated caspase-3 and induce neural cell apoptosis, resulting in cerebral structure changes and neurocognitive disorder (Jafarian et al. 2015; Lin et al. 2016; Takada et al. 2015). In addition, severe sepsis can contribute to hippocampal mitochondrial impairment in experimental models (d’Avila et al. 2008). Magnifico et al. showed that NAD^+^ acts on mitochondrial SIRT3 to prevent axonal caspase activation and axonal degeneration (Magnifico et al. 2013). Consistent with previous studies, this study showed that the number of TUNEL-positive nuclei and the expression of caspase-3 protein in the hippocampus increased in the mice undergoing CLP surgery, suggesting that the possible neuronal apoptosis induced by CLP contributed to neurodegeneration and consequent impairment in spatial learning and memory. This study also found that downregulation of CypD by siRNA infection and SIRT3 overexpression attenuated apoptotic and necrotic types of cell death, and had positive effects in the hippocampus. These in vivo experiments further suggested that an increase in SIRT3 and a decrease in CypD induced neuroprotection, which are mediated by preventing neuronal apoptosis. The mPTP opening causes mitochondrial dysfunction, which can be cut down in light scattering at 540 nm. In this study, mPTP opening decreased in the CypD-si and SIRT3-p groups compared with the CLP group, suggesting that the administration of decreasing CypD and increasing SIRT3 can reverse mitochondrial dysfunction.

Elevated levels of cytokines, such as IL-1β, TNF-α, and IL-6, have been observed in the brain parenchyma and cerebrospinal fluid after sepsis (Jeremias et al. 2015; Takahashi et al. 2014). The neuropathology in SAE is quite similar to that in patients with severe sepsis in terms of elevated levels of proinflammatory cytokines in the brain (Blom et al. 2015). Sepsis-induced alterations in mitochondrial damage were associated with SIRT3 downregulation. SIRT3 activation plays a protective role against mitochondrial damage in the kidney by attenuating oxidative stress, inhibiting the NRLP3 inflammasome, attenuating ROS production, and downregulating IL-1β (Zhao et al. 2016). SIRT3 deletion increases the expressions of IL-1β, TNF, and IL-6 in the inflammation (Lim et al. 2016). It has been proven that SIRT3 decreased in neurodegeneration such as Alzheimer’s disease (Jęśko et al. 2016). This study found that TNF-α and IL-6 decreased in the CypD-si and SIRT3-p groups, which prompted us to hypothesize that these cytokines could be generated in SAE. It implied that the inflammatory factors in hippocampus increased significantly in SAE mice, which is consistent with the findings of the previous studies, and CypD knockdown and SIRT3 overexpression inhibited the expression of these cytokines.

Shulga et al. have shown that the posttranslational modification of CypD plays a role in mPTP regulation (Shulga and Pastorino 2016). The overexpression of SIRT3 in vitro attenuates mitochondrial dysfunction, whereas its knockdown can increase mitochondrial depolarization and subsequently cause cell death. These data observed by Shulga et al. indicate that SIRT3 can inhibit the binding of CypD to adenine nucleotide translocator (ANT) in HeLa cells, and the progression is completed depending on CypD deacetylation by SIRT3 (Bochaton et al. 2015). A previous study provided evidence that SIRT3 plays an important role in mediating neuroprotective effect and its deficiency disrupts the advantageous effects in protecting hippocampal cells in the CA1 and CA3 regions (Weir et al. 2012). SIRT3 can maintain the balance of protein acetylation in mitochondrial neurons, and its deficiency or reduction in mouse models of temporal lobe epilepsy also results in hyperacetylation of CypD (Li et al. 2015). Therefore, SIRT3 protects mitochondrial structure and function by deacetylating mitochondrial proteins including CypD. Since SIRT3 is a deacetylase, its involvement in CypD-dependent modulation of mPTP opening during cognitive impairment was examined in this study. Our findings suggested that SIRT3 prevented excessive mitochondrial damage by deacetylating and subsequently inactivating CypD protein. Moreover, SIRT3 prevented mPTP formation and sustained MMP by inhibiting CypD acetylation and subsequent activation. This study first observed that mitochondrial damage in hippocampus induced by sepsis led to an increased acetylation of CypD. The hyperacetylation of CypD in hippocampus, where its deacetylation reduced, triggered the opening of mPTP, which induced the mitochondrial damage.

This study suggested that the activation of SIRT3 alleviated learning and memory dysfunction induced by sepsis. This effect was most likely associated with the deacetylation of CypD mediated by SIRT3 that inhibited mPTP opening and subsequently attenuated mitochondrial impairment after CLP surgery in mice.

## Conclusions

In conclusion, CypD deacetylation and SIRT3 overexpression in hippocampus attenuated learning and memory dysfunction induced by sepsis. By deacetylating and thereby activating CypD, SIRT3 prevented mitochondrial damage, decreased the expression of inflammatory cytokines, and inhibited neural apoptosis, resulting in neuroprotection against cognitive dysfunction. This study opens new perspectives for developing protective therapies against the occurrence and development of SAE.
